# Understanding never events: developing an ecologically valid behavioral test for left-right confusion during surgery

**DOI:** 10.3389/fcogn.2026.1802120

**Published:** 2026-08-31

**Authors:** Moritz Thaler, Henning Parschau, Janne Carstensen, Lorenz Mühlig, Jonathan Kirner, Hümeyra Altunkaynak, Gabriel Kändler, Rainer Petzina, Sebastian Ocklenburg

**Affiliations:** 1 Department of Psychology, MSH Medical School Hamburg, Hamburg, Germany; 2 Institute for Safety of Patients and Health Professionals (ISPP), MSH Medical School Hamburg, Hamburg, Germany; 3 Institute of Cognitive Neuroscience, Biopsychology, Faculty of Psychology, Ruhr University Bochum, Bochum, Germany

**Keywords:** behavioral test, laterality, left-right confusion, never events, patient safety, spatial cognition, surgery

## Abstract

**Introduction:**

The accurate distinction between left and right is a critical cognitive function. A failure to correctly distinguish left and right during surgery can lead to a so-called Never Event, a preventable medical error that leads to death or severe injury of the patient. Unfortunately, such wrong-site surgeries still happen every year. Thus, understanding left-right confusion during surgery is critical for patient safety. However, current Left-Right Confusion (LRC) tests have low ecological validity for the complex, time-critical demands of a surgical environment. This study introduces the Never Events in Surgery Test (NEST), a novel, ecologically valid behavioural paradigm designed to simulate the cognitive dynamics during surgery.

**Methods:**

Participants (*N* = 108) completed the NEST alongside established LRC tasks: the Bergen Left-Right Discrimination Task (BLRDT), the Left-Right Commands Task (LRCT), and the Left-Right Questionnaire (LRQ).

**Results:**

NEST performance was significantly modulated by the posture of the training doll. Error rates were lowest in the prone position compared to lateral orientations (*p* < 0.05), while the supine position resulted in the shortest reaction times, significantly outperforming both prone and lateral positions (*p* < 0.001) without a significant increase in error rates. Crucially, the study established a cognitive dissociation: while reaction times across tasks were correlated, reflecting a shared component of general processing speed, the error rates from the established LRC measures (BLRDT, LRCT, LRQ) failed to predict the error rate observed in the applied NEST paradigm.

**Discussion:**

The lack of predictive power for errors suggests that LRC susceptibility in applied contexts is an independent, context-specific risk factor for laterality errors not captured by traditional assessments. The findings argue for a paradigm shift in training and screening toward tailored, context-specific approaches.

## 1 Introduction

The accurate distinction between left and right is a complex and critical cognitive function whose failure can lead to significant consequences, particularly in high-stakes processes like performing surgery. The failure to distinguish left and right properly is known as Left-Right Confusion (LRC). LRC is defined as a persistent difficulty in correctly identifying, naming, or distinguishing between left and right sides of a body or objects in space, leading to systematic laterality errors (van der Ham et al., 2021).

Learning to differentiate between left and right is a developmental process spanning several years. While most individuals are capable of labeling their own body parts with the correct spatial orientation by the age of seven (Dellatolas et al., 1998), the ability to accurately distinguish left and right reaches adult levels only around the age of 12 (Benton, 1968). Quantifying the prevalence of LRC reveals that this difficulty is widespread. According to a recent comprehensive study by van der Ham et al. (2021), 14.2% of individuals reported frequently confusing left and right. When objectively measured using experimental tasks, the authors found that 8.4% of participants actually made laterality errors. A significant proportion of the respondents (42.9%) reported using a specific strategy to safely distinguish between left and right.

Regarding the prevalence of LRC, Wolf (1973) provided some of the first empirical data, reporting that 17.5% of female and 8.8% of male participants claimed to frequently confuse left and right during rapid decision-making tasks. Subsequent research by Sholl and Egeth (1981) then explored the underlying mechanisms, suggesting that verbal encoding is strongly associated with LRC. Nevertheless, the majority of knowledge concerning the neurocognitive processes underlying LRC historically stems from clinical studies involving patients affected by Gerstmann Syndrome. Over eight decades ago Gerstmann (1940) proposed a clinical syndrome that was associated with a lesion of the posterior parietal lobe in the dominant (typically left) hemisphere. The syndrome was defined by a tetrad of four symptoms: agraphia (acquired writing impairment), finger agnosia (inability to recognize, name and distinguish fingers), acalculia (loss of ability to perform arithmetic operations) and the inability to distinguish left from right. Beyond this acquired syndrome, an increased prevalence of LRC has been reported across a range of psychiatric, neurodevelopmental and neurological disorders. A recent systematic review by Thaler and Ocklenburg (2025) aimed to comprehensively identify disorders where LRC prevalence is higher than in the general population. Their analysis confirmed that severe impairments in laterality orientation are associated with lesions in parietal brain areas and damage to temporal-parietal-occipital brain regions due to conditions such as Alzheimer's disease. Furthermore, evidence for developmental impairments of left-right discrimination was provided by studies on developmental topographical disorientation. Ultimately, the persistence of LRC across healthy and clinical populations confirms its status as an important vulnerability, particularly in high-risk environments like a surgical setting.

Failure to correctly identify the correct side to perform a medical procedure on remains a persistent, albeit infrequent, safety concern in hospital. Wrong-site surgery is classified as a so-called Never Event (NE), i.e., completely avoidable, severe incidents within health institutions that lead to patient harm, up to and including death (Koleva, 2020).

Left-right confusion is a specific kind of NE that potentially results in the amputation of the wrong body part (Bowman et al., 2023; Gormley et al., 2018; Hoppes et al., 2013). A recent study analyzed NE NHS data in England from 2012 to 2020 and revealed that out of 797 surgical NEs, 84 happened due to confusion between left and right (Omar et al., 2021). This suggests that LRC is a causal factor for at least a small proportion of surgical NE. Although various organizations (e.g., National Patient Safety Agency) have attempted to prevent these by using body markings or checklists, laterality errors continue to occur (Ocklenburg et al., 2024; Panesar et al., 2011). Understanding the underlying cognitive mechanisms of LRC is therefore essential to mitigating the risk of confusing left and right during surgery.

In the literature, objective measures of LRC are sparsely reported. Most established objective tests rely on distinguishing left and right body parts, with hands being the most frequent choice. Early objective tasks utilized schematic drawings of lateralized bodies or hands (Brandt and Mackavey, 1981; Leli et al., 1982; Ratcliff, 1979). A highly similar methodology was later adopted by Hirnstein et al. (2009) and Hjelmervik et al. (2015), who substituted drawings with photographs of hands in various orientations. Alternatively, Vingerhoets and Sarrechia (2009) described a more language-based task where participants made finger presses in response to the written words “left” and “right” appearing on a screen.

These methodologies reflect the essential theoretical decomposition of LRC into two primary, distinct components within cognitive psychology and neuropsychology: spatial-cognitive ability, which involves mental spatial transformations and verbal-semantic ability, which relies on language processing (Ocklenburg et al., 2011; Ofte, 2002; Sholl and Egeth, 1981). The Bergen Left-Right Discrimination Test (Ofte and Hugdahl, 2002) is a standardized objective measure primarily targeting the spatial-cognitive component of LRC. The test uses schematic stick figures, viewed from various angles and perspectives, forcing participants to mentally rotate the figures to correctly identify their left or right hand. The Left-Right Discriminations Task (Hirnstein et al., 2009) is an objective, performance-based task that assesses the verbal-semantic component of LRC. Participants follow verbal commands concerning their own left and right body parts (e.g., “Lift your left hand!”) and performance is compared between simple and complex command conditions. Finally, the Left-Right Questionnaire (Jordan et al., 2006) provides a subjective context. It consists of eight items rated on a five-point scale. Previous work, such as that by Rigal (1994), highlighted the complexity of this dissociation. While this cognitive decomposition has been crucial for theoretical understanding, it remains unclear how well these basic components predict errors in complex, time-critical operational settings. To capture the multi-faceted nature of LRC, the current study utilized the three established measures: the Bergen Left-Right Discrimination Task, the Left-Right Commands Test and the self-rate Left-Right Questionnaire.

However, these established tests often fail to capture the real-world cognitive load people experience in stressful situations that require left right discrimination. Specifically, they possess low ecological validity regarding the time-critical, multi-perspective demands of a realistic setting (Ocklenburg et al., 2024). Ecological validity refers to the extent to which the findings of a study can be generalized to real-life settings (Bronfenbrenner, 1979); in this context the tasks do not accurately reflect the complexity of a surgical environment. Therefore, a major limitation in current research is the lack of a standardized behavioral instrument capable of assessing LRC performance under conditions that simulate the cognitive load of a surgical environment. Left right discrimination in applied contexts may involve additional spatial and embodied transformation processes not captured by traditional LRC tasks. Spatial transformation refers to the cognitive process of mentally rotating or translating objects in space, while embodied transformation involves simulating the spatial relationship of the patient's anatomy from the surgeon's own first-person perspective, which can be complicated by factors like awkward body position (Shepard and Metzler, 1971).

This gap presents a critical research need. Current methodologies fail to isolate and measure the specific type of LRC error, that contributes to wrong-site surgery. The inability to objectively assess LRC performance under high-pressure, multi-perspective conditions hinders the development of effective, targeted training protocols and preventive strategies for high-stakes environments. Therefore, the primary motivation for this research is to develop and validate a behavioral instrument that bridges this ecological validity gap, allowing for a more accurate assessment and mitigation of LRC risk in professional medical settings.

The present study addresses this gap by introducing the Never Events in Surgery Test (NEST), a novel paradigm developed to simulate the cognitive dynamics of an operating environment. The NEST requires participants to integrate auditory instructions with the current spatial orientation of a professional cardiopulmonary resuscitation training doll commonly used in medical training under time pressure. Thereby, it yields a better model of the complexity of a surgical environment than a standard computerized LRC task. To contextualize NEST performance within established left–right confusion assessment traditions, participants additionally completed three benchmark tasks: the Bergen Left-Right Discrimination Task (BLRDT), the Left-Right Commands Task (LRCT), and the Left–Right Questionnaire (LRQ), replicating a previous study by Ocklenburg et al. (2011). This approach enabled direct comparison of NEST with well-validated cognitive measures of spatial, verbal and subjective left–right discrimination, allowing us to clarify whether classical LRC components generalize to an operational context and to evaluate the ecological sensitivity of our new test. Our hypotheses mirror these objectives.

First, to ensure comparability with established literature, we aimed to replicate key effects in the established tasks according to Ocklenburg et al. (2011). We expected to confirm the influence of spatial complexity in the BLRDT, observing a monotonic increase in error rates with stimulus complexity. Similarly, in the LRCT, we hypothesized a significant main effect of stimulus Type, with performance decreasing as complexity increases.

Second, to validate the NEST, we hypothesized that performance would vary significantly based on the posture of the cardiopulmonary resuscitation training doll. Specifically, positions requiring more complex spatial adjustment (e.g., lateral positions) should lead to increased errors and reaction times, confirming that LRC is context-dependent (McKinley et al., 2015). Notably, prior studies have shown that participants make fewer errors when stimuli are congruent with their own bodily orientation. This effect suggests that when the visual information aligns with the observer's body centered perspective, accurate left-right discrimination is facilitated (Ofte and Hugdahl, 2002). In addition, studies indicate that minimizing frequent shifts in perspective, especially when the task remains anchored to a familiar, body-centered reference frame, also leads to improved performance. Collectively, these findings support the notion that congruence (alignment between stimulus and observers innate spatial reference) is crucial for an accurate distinction between left and right (Ofte and Hugdahl, 2002).

Furthermore, given previous research on sex differences in spatial tasks, we anticipated that the NEST task would reveal sex differences in performance, potentially interacting with training doll posture (Gormley et al., 2008; Hirnstein et al., 2009; Ocklenburg et al., 2011; Ofte and Hugdahl, 2002).

Crucially, our central hypotheses focused on cross-task generalizability of performance metrics and on the predictive power of basal LRC metrics on the applied NEST task. To test for the expected generalizability of processing speed and the dissociation of error mechanisms across the three tasks, we first examined the bivariate correlations between all reaction times (RT) and error components of all tasks. We predicted that NEST reaction time would show significant correlations with and would be significantly predicted by the RT components of the BLRDT and LRCT, reflecting a shared component of general processing speed (Jensen, 2006). However, we hypothesized that the NEST error rate would show non-significant correlations with and would not be significantly predicted by the error rate of the BLRDT and LRCT or results of the LRQ. This expected lack of shared variance is based on the premise that basal LRC paradigms only measure simple, static laterality discrimination, while NEST is designed to specifically capture applied errors resulting from high cognitive load and frequent shifts in the spatial reference frame. These applied errors involve complex spatial and embodied transformation processes that are not present in the established tests. This expected dissociation (RT correlation but non-significant error correlation) would thus demonstrate that the demands of LRC in an applied context measure a largely independent risk factor not captured by established basal LRC paradigms.

By testing these hypotheses, we seek to determine if LRC in an applied context is a standalone risk factor and how its error rate is contextually modulated, providing critical insight for safety protocols in surgical training and practice. Ultimately, the validation of a high-fidelity instrument with improved ecological validity will enable the targeted identification and mitigation of specific LRC risks in the operating theater, thereby directly addressing the persistent clinical challenge of wrong-site surgery.

## 2 Method

### 2.1 Participants

Since this study aimed at establishing a new task, no previous effect sizes to properly conduct power analysis existed. Therefore, we based the sample size on the sample sizes in previous studies on LRC. The initial sample comprised 120 participants. Data was first screened for completeness and consistency across all data files. During the data merging and anonymization process, 12 participants were excluded due to technical errors.

The final sample included *N* = 108 participants (i.e., 39 males, 69 females). The mean age was *M* = 27.7 years (*SD* = 9.58). No participants reported history of neurological or psychiatric disorders. Written informed consent was obtained from all participants, and the study was conducted in accordance with the Declaration of Helsinki. The study protocol was approved by the Ethics committee at MSH Medical School Hamburg (IRB-number: MSH-2024/371).

### 2.2 Procedure

The experimental session was conducted individually. Participants completed the three tests in a fixed, non-randomized order: BLRDT, followed by LRCT, and finally the NEST. This fixed sequence ensured that the basal cognitive predictors were measured prior to the complex, applied task.

The test session began with participants providing informed consent and receiving a unique participant code. Participants were then seated at a computer to complete the LRQ (German short version). Subsequently, the three objective behavioral tasks were administered consecutively to prevent potential stereotype activation effects. The first task was the seated, computerized BLRDT. This was immediately followed in the test area by the LRCT and the novel NEST. Both the LRCT and NEST utilized video recording for objective *post-hoc* error scoring. The session concluded with participants completing a final back-reference questionnaire.

### 2.3 Data processing

All raw data files were anonymized using a pre-generated key table. Performance variables were aggregated from trial-level data (reaction time and error rates) for each test.

For the multiple regression analysis (testing H4), participants with missing data in any of the six performance outcome variables were excluded to ensure the use of Complete Cases. This resulted in a final analytical sample size of *N* = 101 for the regression models.

#### 2.3.1 Inter-rater reliability

To assess the reliability of the scoring procedure for the NEST and LRCT, a subset of 11 participants (randomly selected from the full sample) were independently re-scored by a second rater using video recordings of the experimental sessions. Inter-rater agreement was high (*M* = 98.23%, range: 96.5%−99.5%), indicating excellent reliability of the scoring procedure.

### 2.4 Materials

To provide a comprehensive assessment of LRC, the study employed three established measures: the LRCT, the BLRDT, and LRQ, along with the novel NEST. All computerized tasks were programmed and presented using *PsychoPy* (Version 2023.2.3; https://www.psychopy.org; Open Science Tools, 2025). All voice commands were generated using the text-to-speech program *Balabolka* (Version 2.15; https://www.cross-plus-a.com/de/balabolka.html; Ilya, 2025).

#### 2.4.1 Never Events in Surgery Test (NEST)

The NEST was a novel, custom-generated task designed to measure LRC in an applied context within a spatial-operational context, requiring participants to perform actions on an external reference object. The test utilized a life-sized, anatomically correct cardiopulmonary resuscitation training doll placed in a lying position on a medical examination table in the testing room. Auditory commands, instructing the participant to physically point to a specific, lateralized body part on the training doll, were generated using the software *Balabolka* and delivered via the experimental software *PsychoPy* (see Ch. 2.4).

The experiment manipulated two independent spatial factors:

1 Training Doll Posture: The cardiopulmonary resuscitation training doll was physically adjusted by the experimenter to create four primary conditions: Supine (on its back), prone (on its stomach), left lateral (on its left side), and right lateral (on its right side).

2 Participant Start Position (starting position 1 - 4): A set of four pre-defined starting positions determined the participant's position relative to the training doll the moment the auditory command was presented. This factor introduced an additional layer of spatial complexity and minimized the bias from a fixed viewing angle. The starting position was randomized after each trial and the distance between the training doll and the starting position remained constant across all trials and conditions.

Each of the four conditions consisted of 50 trials, presented in a pseudo-randomized order. Five neutral trials (e.g., pointing to the navel or spine), which were irrelevant to lateralization, were included in each block as control measures and subsequently removed from the analysis, resulting in a total of 180 critical trials (4 conditions x 45 critical trials). After every block of 50 trials, the training doll was repositioned before the next trial block started.

The procedure is best illustrated by a typical trials example: Following the participants execution of the previous command, the next randomized starting position was communicated via the *PsychPy* software (e.g., “Starting position three”). The participant was required to immediately move to this new position. Once the participant was confirmed to be situated at the designated starting point, the experimenter manually triggered the subsequent verbal command via the software (e.g., “Tap the left knee.”) The participant then attempted to execute the command on the training doll as quickly and accurately as possible. The cycle concluded as the software communicated the next randomized starting position. Crucially, all verbal output (both for the starting position announcements and the operational commands) were generated using the *PsychoPy/Balabolka* software to ensure standardized articulation and timing.

**Figure 1:**
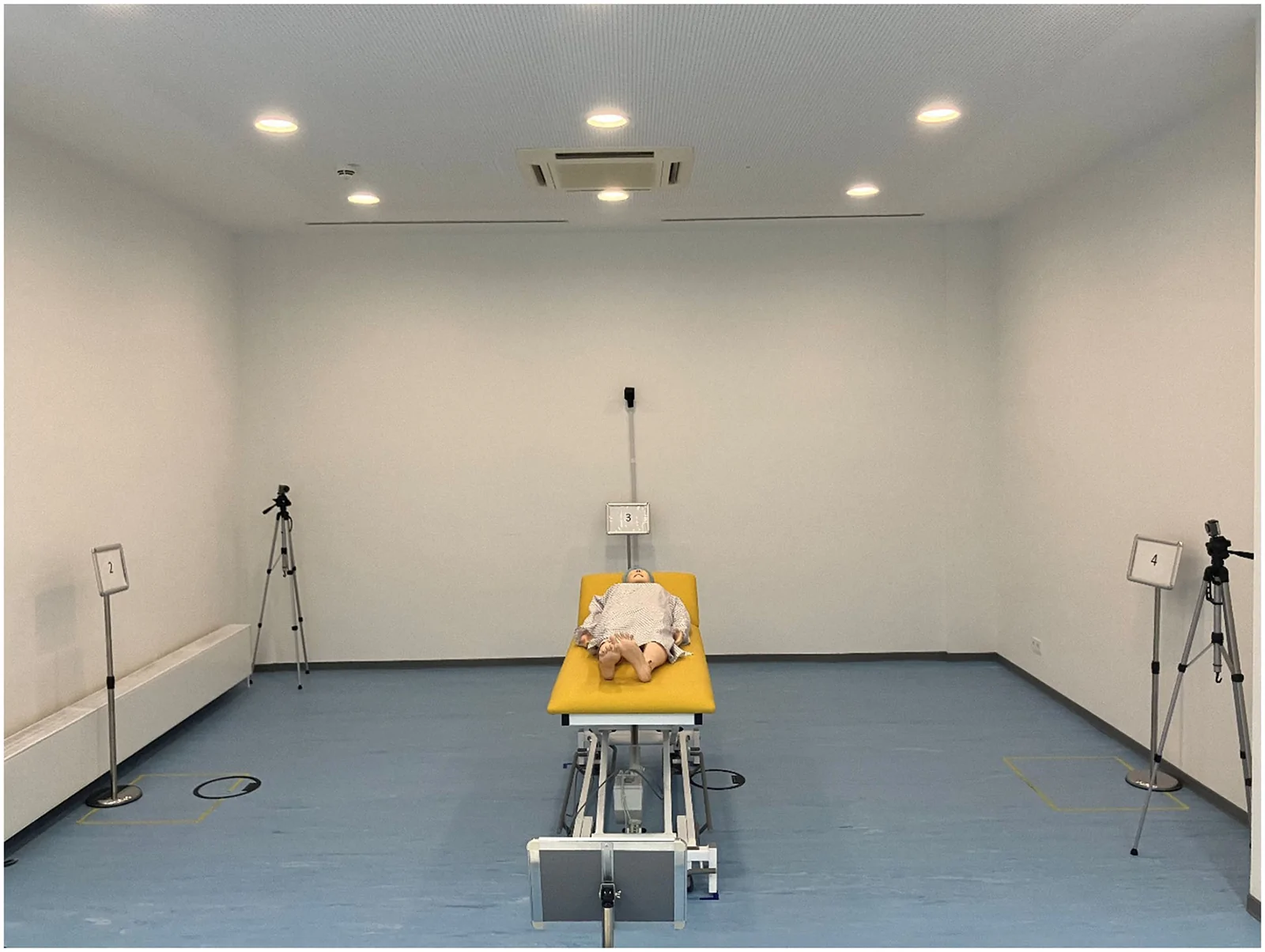
Photo presentation of the testing room: the anatomically correct cardiopulmonary resuscitation training doll can be seen, laying on the medical examination table in the supine condition. In the back left corner of the testing room one of the cameras used to record the NEST trails can be seen.

**Figure 2:**
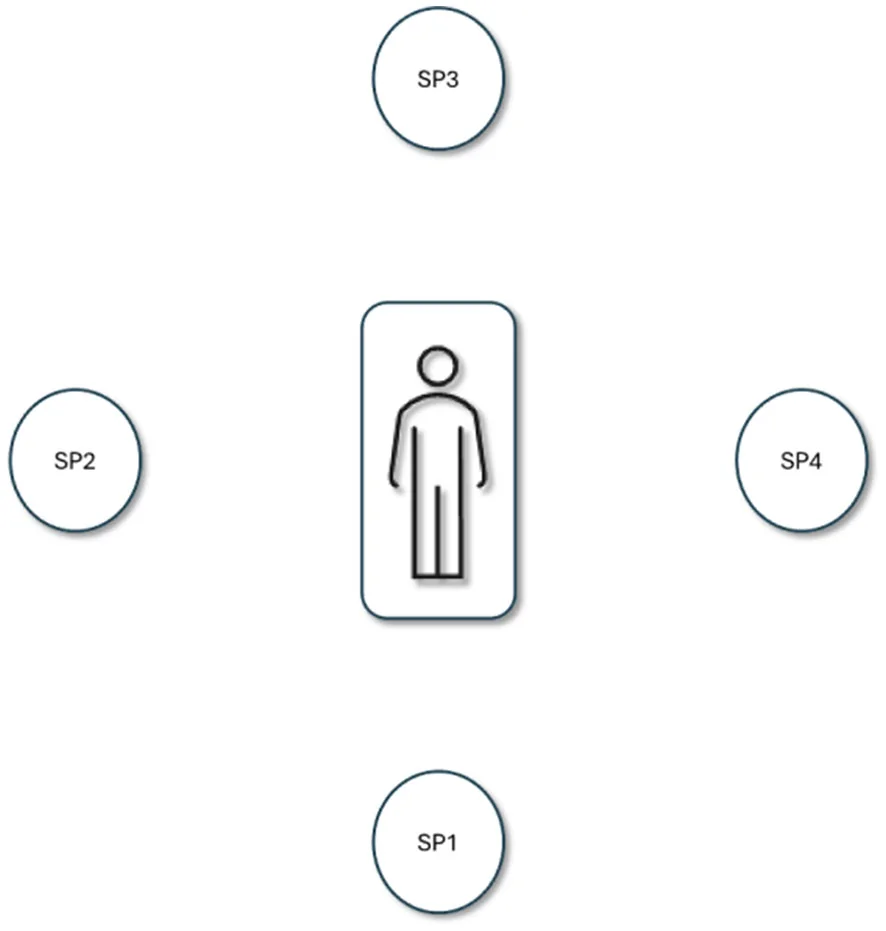
Schematic illustration of the experimental setup for the NEST task. The participant moves between four starting positions between each trial (SP1-SP4), surrounding the patient mannequin. SP1 served as the initial starting point for each condition.

**Figure 3:**
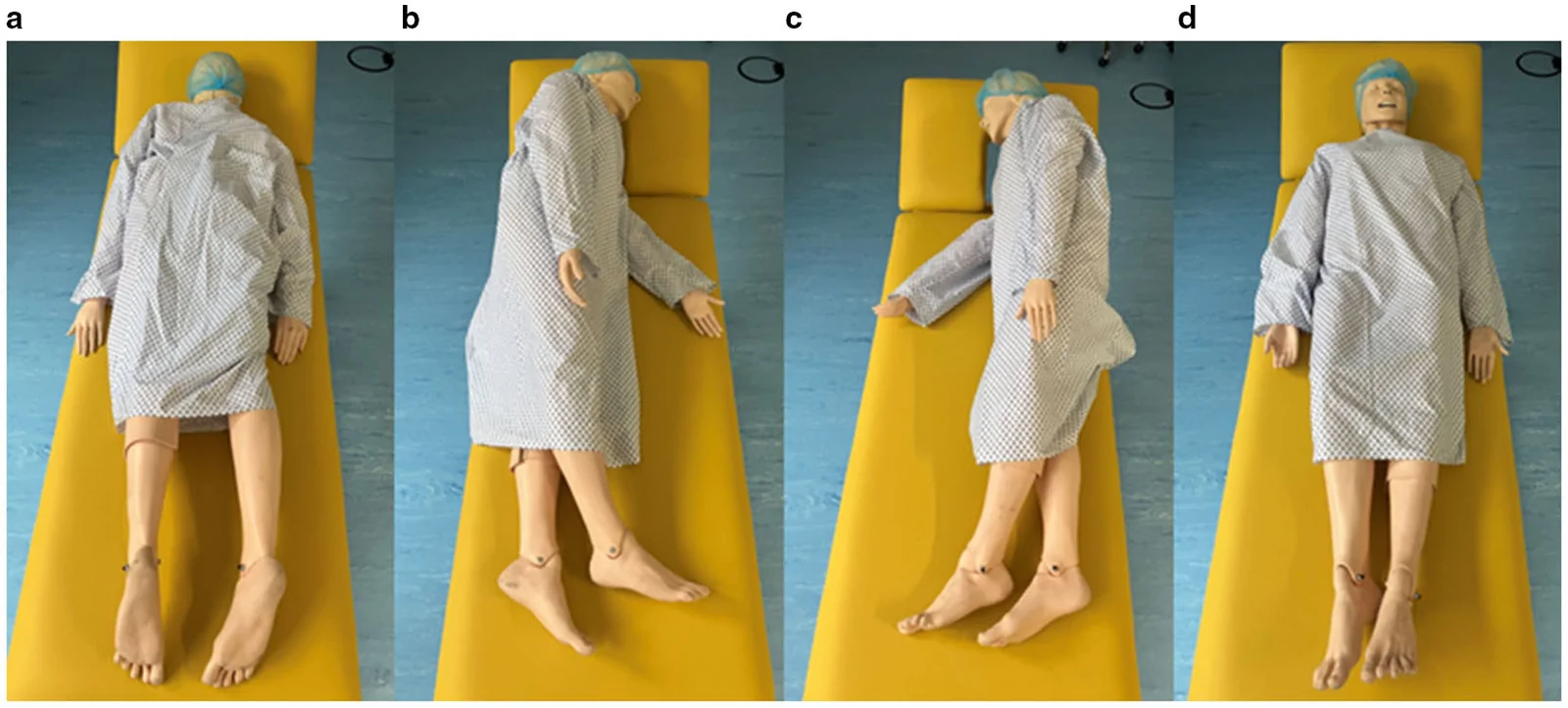
Photo report of the different mannequin positions: prone (a), left side (b), right side (c) and supine (d).

The dependent variables were the LRC error count (binary 0/1 per trial) and the corresponding reaction times (RTs). An error was scored if the participant correctly performed the required action but confused the lateralization relative to the training doll. Any trial for which the response time exceeded 7 s was excluded from the final error and RT analysis. Both error rates and reaction times were analyzed based on the main condition (training doll posture) and the starting position of the participant. The experimenter manually recorded the participant's response (correct/incorrect) directly into the software. While reaction times were logged automatically via PsychoPy into the data file at the end of each trial, the trial termination itself was triggered manually by the experimenter, who pressed a designated key the exact moment the participant executed their physical pointing response. Consequently, the recorded reaction times include a minor, systematic component of experimenter latency. The NEST setup and experimental procedure are illustrated in Figures 1–3, including the clinical simulation room (Figure 1), the spatial arrangement of the stimulus positions (Figure 2), and the different mannequin positions used during the test (Figure 3).

#### 2.4.2 Bergen Left-Right Discrimination Test (BLRDT)

The BLRDT (Ofte, 2002, adapted by Ocklenburg et al., 2011) was administered using a computerized version, replicating the methodology of the source paper and using the original stimuli. The task consisted of 96 line drawings of a stick figure presented either from a back view (black head) or a front view (white head). One of the two hands of the figure was highlighted in red and participants had to quickly decide whether the label (‘R' or ‘L') presented below the figure matched the highlighted hand. The label was correct in 50% of the trials. This replication employed a standard horizontal arrangement of the response keys. The main dependent variables were the LRC error Rate (percentage of incorrect matches) and the RTs for correct responses.

#### 2.4.3 Left-Right Commands Task (LRCT)

The LRCT was a replication of the task described by Hirnstein et al. (2009). The original German text templates were used, but the acoustic files were generated using the text-to-speech program Balabolka with the german Microsoft “Hedda” voice. Commands were divided into three 20-trial conditions: control, simple (single limb movement), and complex (crossed limb movements). A total of 60 standardized verbal commands were presented acoustically.

In the beginning, participants were seated with their hands resting on their knees (starting position) before being instructed to execute the command and return to the starting position before the next command appeared after 750 ms. LRC was defined strictly as the confusion of the left and right side while correctly performing the required action. Participants were video recorded to ensure accurate *post-hoc* scoring of LRC errors by the experimenter. The dependent variable was the LRC error percentage for the simple and complex command conditions.

#### 2.4.4 LRC self-rating questionnaire

Subjective susceptibility to LRC was measured using the german short version of the self-rating questionnaire (Jordan et al., 2006; adapted by Hirnstein et al., 2009).

The questionnaire comprised eight items categorized into: four LRC-Items (specific to left-right judgments) and four DIR-Items (relating to general directional abilities). Participants rated each item on a five-point Likert scale, ranging from ‘no problems at all' (1) to ‘severe problems' (5). This scale and the item categories were maintained to allow for comparison with previous research. Scores were aggregated separately for the LRC and DIR categories.

### 2.5 Statistical analysis

Statistical analyses were performed using R-Studio (Version 2025.09.0 + 387; https://posit.co/downloads). The significance level was set to α = 0.05.

## 3 Results

### 3.1 Validation of basic LRC Tasks (Replication)

#### 3.1.1 BLRDT

A 3 x 2 x 2 mixed ANOVA was conducted on the BLRDT performance metrics with Arm Position (no arm, one arm, both arms crossing the midline) and View (back view, front view) as within-participants factors, and sex as a between-participants factor.

For LRC Error Rates, a significant main effect was found for Arm Position, *F*_(2, 214)_ = 7.88, *p* < 0.001, ηG^2^ = 0.020, and for the Arm Position x View interaction, *F*_(2, 214)_ = 7.92, *p* < 0.001, ηG^2^ = 0.003. The main effect of sex was not significant [*F*_(1, 107)_ = 0.59, ns, Cohen's *d* = −0.135, 95*% CI* [−0.530, 0.259]). Post hoc tests on the effect of the arm position revealed that error rates were significantly higher when both arms crossed the midline (“both_arms”) compared to the “one_arm” (*p* < 0.001) and “no_arm” (*p* = 0.036) conditions, though no significant difference existed between “no_arm” and “one_arm” (*p* = 1.0). All other main effects and interactions were non-significant (all *p* > 0.05, all ηG^2^ < = 0.003).

For RTs, a significant main effect of Arm Position was observed, *F*_(2, 206)_ = 56.20, *p*<*0.001*, η*G*^2^ = *0.0*49. *Post hoc* comparisons with Bonferroni adjustment showed that RTs were significantly longer when both arms crossed the midline (*M* = 4.33 s, *SD* = 1.83) compared to one arm (*M* = 3.89 s, *SD* = 1.83, *p* < 0.001) and no arms crossing (*M* = 3.41 s, *SD* = 1.12, *p* < 0.001). Crucially, RTs for the “one arm crossing” condition were also significantly longer than for the “no arms crossing” condition (*p* < 0.001).

**Figure 4:**
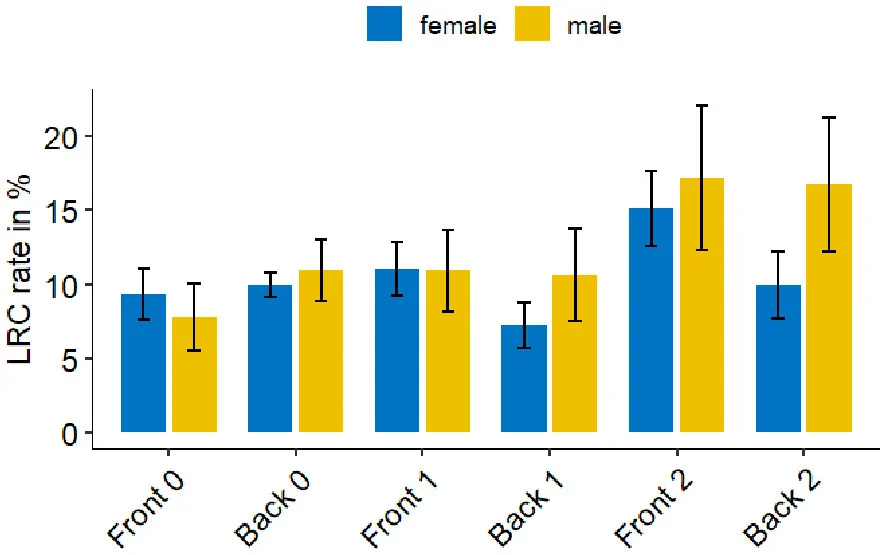
Sex specific LRC rate in % for BLRDT.

Additionally, a significant main effect of View was found, *F*_(1, 103)_ = 20.29, *p* < 0.001, η*G*^2^ = 0.031, as well as a significant Arm Position x View interaction, *F*_(2, 206)_ = 4.37, *p* = 0.014. Furthermore, a three-way interaction between Arm Position, View, and Sex was observed, *F*_(2, 206)_ = 3.36, *p* = 0.037. The main effect of sex was not significant, *F*_(1, 103)_ = 0.09, *p* = 0.771, ηG^2^ < 0.001. Sex-specific LRC rates for the BLRDT are shown in Figure 4.

#### 3.1.2 LRCT

A repeated-measures ANOVA revealed a significant main effect of condition on reaction times (*F*_(2, 212)_=1197.51,*p* < 0.001,ηG^2^ = 0.605), while neither sex, *F*_(1, 106)_ = 0.01, *p* = 0.932, nor the sex-by-condition interaction reached significance (*p* = 0.911, ηG^2^ < 0.001). Mauchly's test indicated no violation of sphericity, and both Greenhouse-Geisser and Huynh-Feldt corrections confirmed the robustness of this effect. *Post hoc* pairwise comparisons with Bonferroni adjustment showed that reaction times were significantly faster in the neutral condition compared to both simple and complex conditions (neutral vs. simple: *p* < 0.001; neutral vs. complex: p < 0.001), and reaction times differed significantly between simple and complex as well (*p* < 0.001).

**Figure 5:**
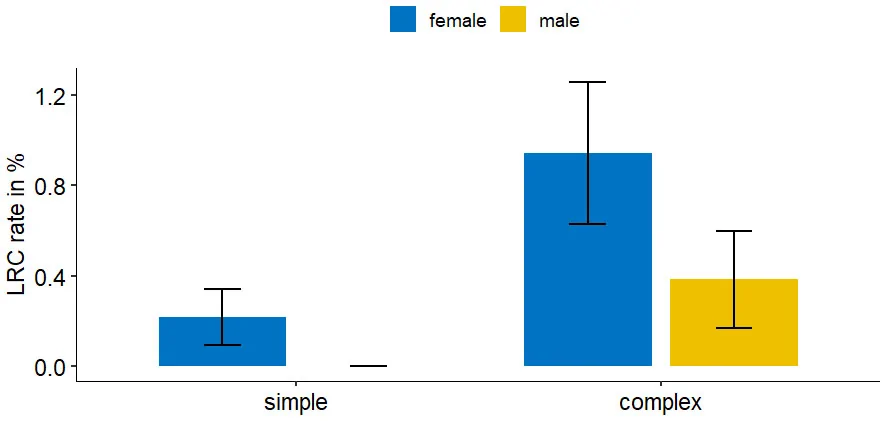
Sex specific LRC rate in % for LRCT.

For error rates, a significant main effect of condition was observed, F _(2, 212)_ = 5.99, *p* = 0.003, ηG^2^ = 0.037 with *post hoc* tests indicating that errors were less frequent in the neutral condition compared to complex (*p* = 0.011). No significant differences were found for neutral vs. simple (*p* = 0.601) or simple vs. complex (*p* = 0.113). All analyses were conducted averaging over sex. A visualization of the sex-specific error rates for the LRCT is shown in Figure 5.

#### 3.1.3 LRQ

The LRQ was analyzed across its two dimensions, LRC and DIR (directional questions), using the average rating per item (score 1-5). Due to significant non-normality in most scores, non-parametric tests were employed. Consistent with original findings, a significant difference between men and women was observed across the self-rated scores. On average, women (2.55 ± 0.75) reported significantly greater overall confusion (across both dimensions) than men (2.04 ± 0.49). This difference was highly significant within the LRC dimension (*W* = 1913, *p* < 0.01), where women (2.28 ± 1.14) reported higher LRC scores than men (1.49 ± 0.70). The DIR dimension showed a marginal, non-significant difference between sexes (W = 1649, p = 0.051). Furthermore, a Wilcoxon signed-rank test comparing the two dimensions across all participants revealed a highly significant difference (*V* = 841, *p* < 0.01). Participants reported significantly higher scores for DIR items (2.74 ± 0.58) compared to LRC items (1.99 ± 0.96).

### 3.2 NEST

#### 3.2.1 Internal consistency

The split-half reliability of the NEST test was assessed by dividing the trials into two halves for each participant and calculating the total number of errors per half. The correlation between the two halves was *r* = 0.712, indicating a strong positive relationship. Using the Spearman-Brown correction to adjust for test length, the reliability estimate was 0.832.

#### 3.2.2 Training doll posture

**Figure 6:**
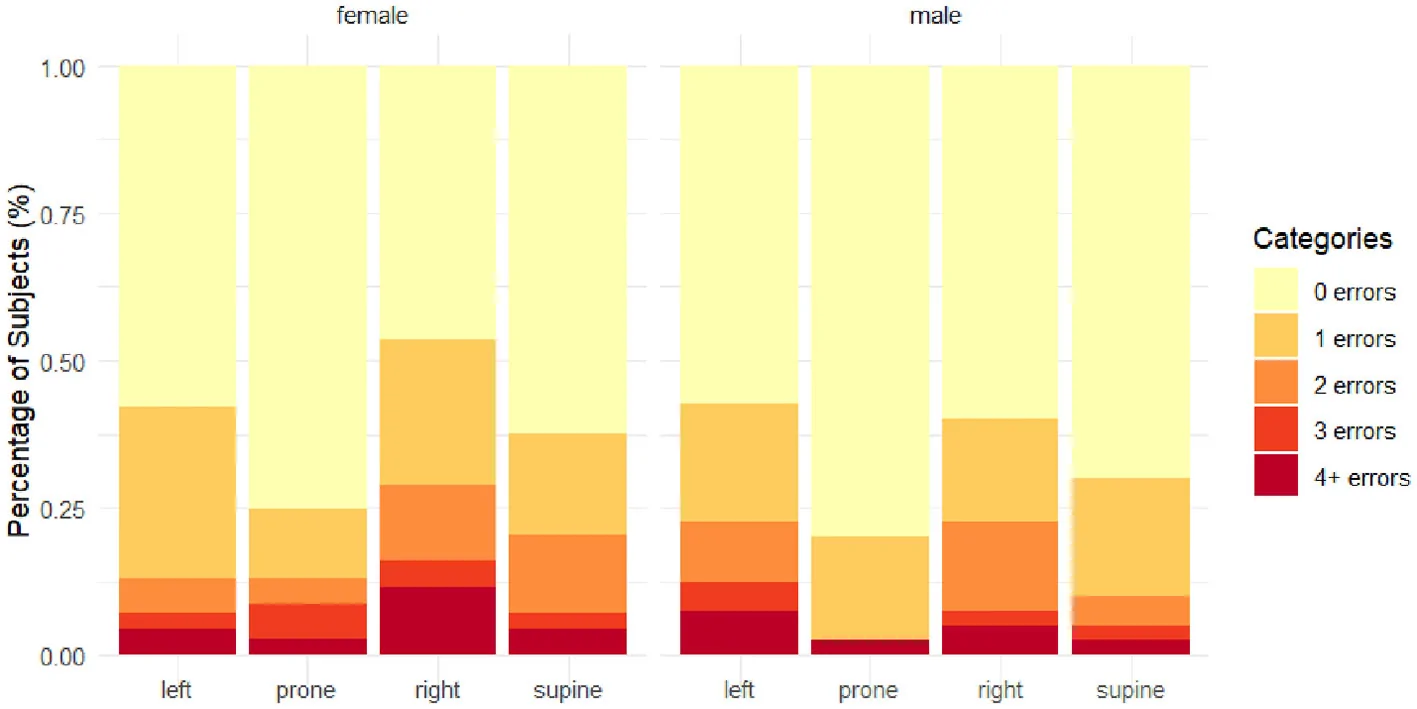
Sex specific LRC error percentage for NEST.

The analysis of errors across conditions showed a significant difference, as indicated by the Friedman test, χ^2^ (3) = 11,23,*p* = 0.011, Kendall's W = 0.055. *Post-hoc* Dunn tests with Bonferroni correction revealed that error rates in the prone condition were significantly lower than in the left (adjusted *p* = 0.018, *d*= −0.11) and right conditions (adjusted *p* = 0.044, d= −0.22), and in the supine condition compared to right (adjusted *p* < 0.001, d = −0.33). No significant differences were found between left and right conditions or between prone and supine conditions after correction. Sex-specific LRC error percentages for the NEST depending on the mannequin position are shown in Figure 6.

Reaction time analysis using the Kruskal-Wallis test showed a highly significant effect of condition, χ^2^ (3) = 182.48,*p* < 0.001,ε^2^ = 0.010. *Post-hoc* Dunn tests with Bonferroni adjustment demonstrated that reaction times were significantly shorter in the supine condition (*M* = 3.96s, *SD* = 1.34s) compared to prone (*M* = 4.41 s, *SD* = 1.62 s), left (*M* = 4.79 s, 1.76s), and right conditions (*M* = 4.43 s, *SD* = 1.64 *s*) (all adjusted *p* < 0.001). Additionally, reaction times in the left condition were significantly longer compared to prone (p < 0.001) and right conditions (*p* < 0.001). There was no significant difference between prone and right conditions.

#### 3.2.3 Sex and interaction effects

Welch's two-sample *t*-test indicated no significant difference in NEST error rates between males (*M* = 2.27) and females (*M* = 3.07), (102.39) = −0.92, *p* = 0.361, Cohen' s d = 0.05 [−0.34, 0.45], 95% CI −2.54, 0.93. The mean error rates were approximately 2.27 for males and 3.07 for females, indicating a small, non-significant tendency for females to make more errors.

A two-way ANOVA assessing reaction times revealed a significant main effect of condition, F _(3, 411)_ = 10.38, *p* < 0.001, but no significant main effect of sex, F _(1, 411)_ = 1.21, *p* = 0.273, nor a sex x condition interaction, F _(3, 411)_ = 0.16, *p* = 0.921. *Post-hoc* comparisons confirmed no significant pairwise differences in reaction times between males and females within any condition (all p > 0.05).

Together, these results indicate no statistically significant sex differences in error rates or reaction times in the NEST test, while reaction times vary by task condition regardless of sex.

### 3.3 Correlational and predictive analysis

#### 3.3.1 Correlations

Spearman rank correlations were computed to assess the relationships between error rates in the NEST test and those in the LRCT and BLRDT tests. A small but significant positive correlation was found between NEST and LRCT error rates, ρ = 0.22, p = 0.023, as well as between NEST and BLRDT error rates, ρ = 0.26, p = 0.006. These results indicate a modest monotonic association of error propensity across these cognitive tasks.

In terms of reaction times, Spearman's rank correlation analyses showed a strong and significant positive association between reaction times in the NEST and LRCT tests (ρ = 0.59,*p* < 0.001). This indicates a robust monotonic relationship, with individuals exhibiting longer reaction times in LRCT also tending to have longer reaction times in NEST. Conversely, the correlation between NEST and BLRDT reaction times was weaker and not statistically significant (ρ = 0.16, *p* = 0.098).

#### 3.3.2 Multiple regression

A multiple linear regression was conducted to examine whether error rates and reaction times from the LRCT and BLRDT tests predicted performance in the NEST test. The model predicting NEST error rates was not significant, F _(6, 94)_ = 0.58, *p* = 0.746, accounting for only 3.6% of the variance (adjusted R^2^=-0.026). None of the predictors (LRCT_errors_, BLRDT_errors_, LRCT_RT_, BLRDT_RT_, LRQ_LRQ_ or LRQ_DIR_) significantly contributed to predicting NEST errors (all *p* > 0.05). The predictors of NEST error rates and mean reaction times are presented in Table 1.

**Table 1:** Predictors of NEST error rates and mean reaction times.

Predictors	Model 1: NEST Error Rate		Model 2: NEST RT	
	*β(estimate)*	*p-value*	*β(estimate)*	*p-value*
(Intercept)	0.03	0.996	0.82	0.088
objective measures (LRCT & BLRDT)				
LRCT (errors)	1.97	0.197	−0.00	0.983
BLRDT (errors)	0.00	0.966	0.00	0.960
LRCT RT (mean)	−0.04	0.985	1.12	< 0.001^*^
BLRDT RT (mean)	0.47	0.311	0.05	0.137
subjective measures (LRQ)			(Not included in Model 2)	
LRQ_LRC	−0.04	0.786	-	-
LRQ_DIR	0.14	0.600	-	-

Model 1: F _(6, 94)_ = 0.58, p = 0.746, ${\text{R}}_{adj}^{2}$ = −0.026; Model 2: F _(4, 96)_ = 14.35, p < 0.001, ${\text{R}}_{adj}^{2}$ = 0.348; LRCT, Left-Right Commands Task; BLRDT, Bergen Left-Right Discrimination Test; LRQ, Left-Right Questionnaire; LRQ_LRC, Left-Right Questionnaire LRC score; LRQ_DIR, Left-Right Questionnaire directional score; RT, reaction time. One observation was excluded from Model 1 due to missing values. For RT predictors and dependent variables, only correct trials were included. ^*^p < 0.001.

Despite the small but significant correlations between NEST error rates and both LRCT and BLRDT, the shared variance remains minimal. Specifically, LRCT accounts for only 5.1% (*R*^2^ = *0.051*) and BLRDT for 5.6 % (*R*^2^ = *0.056*) of the variance of NEST errors. This indicates that NEST performance is largely independent of traditional LRC measures, with over 94% of its variance unexplained by these established tests.

In contrast, the model predicting mean NEST reaction times was highly significant, F _(4, 96)_ = 14.35,*p* < 0.001, explaining 37.4% of the variance (adjusted R^2^=0.348). Here, only the mean LRCT reaction time emerged as a significant predictor (β = 1.12,*t* = 6.95, *p* < 0.001), while other predictors were not significant (all *p* > 0.5).

Another multiple linear regression was conducted to predict NEST error rates from the scores of the self-rating questionnaire LRQ (using LRC and DIR scores). The model was not significant, *F* (2, 102) = 0.30, *p* = 0.743, with an adjusted *R*^2^ of −0.014. Neither sum_lrc (β = 0.06, *p* = 0.651) nor sum_dir (β = 0.09, *p* = 0.711) were significant predictors of NEST error rates.

## 4 Discussion

The present study aimed to resolve methodological inconsistencies in the literature regarding the assessment of LRC by comparing two established LRC tasks (BLRDT & LRCT) with a novel, ecologically valid, operation-oriented LRC test (NEST). Our results confirm the core hypothesis of a cognitive dissociation between the basal, symbolic components of LRC and its performance in an applied, real-world context.

### 4.1 Summary of key findings

This study yielded four principal findings. First, we successfully replicated the core effects in the basal LRC tasks, observing the expected increases in errors and RTs with escalating spatial complexity in the BLRDT and verbal complexity in the LRCT (H1). Second, the NEST demonstrated high ecological sensitivity, showing that performance was significantly modulated by the training doll posture: while error rates were lowest in the prone condition, the supine position elicited the shortest reaction times, thus validating the task's real-world relevance (H2). Third, contrary to expectations based on prior literature (Ocklenburg et al., 2011; van der Ham et al., 2021), we found no significant sex differences in either NEST errors or RTs (H3). Crucially, the fourth and central finding established a cognitive dissociation: while the RT between LRCT and NEST were strongly correlated, reflecting a shared component of general processing speed, the error rates from the established basal measures (BLRDT, LRCT, and the LRQ self-rating) failed to predict the error rate observed in the applied NEST paradigm. This lack of predictive power for errors suggests that LRC in applied contexts represents an independent, context-specific risk factor not captured by traditional assessments (H4). These results further underscore the assumption that LRC is fundamentally complex in nature and depends on many cognitive operations (Hjelmervik et al., 2015).

### 4.2 Replication and validation of established LRC measures (H1)

The results from the Bergen Left-Right Discrimination Test (BLRDT), the Left-Right Commands Task (LRCT) and Left-Right Questionnaire (LRQ) successfully replicated established findings (Ocklenburg et al., 2011; Ofte, 2002) in the cognitive processing of LRC, thereby confirming the construct validity of the traditional tasks used.

In the BLRDT, we observed the expected significant increase in both error rates and RTs with increasing stimulus complexity, specifically in the condition with both arms crossing the midline (complex condition). This aligns with the understanding that the BLRDT primarily assesses the spatial-cognitive component of LRC, where mental perspective-taking and rotation processes increase cognitive load and processing time (Ocklenburg et al., 2011). However, the replication was not complete regarding all demographic variables. Study I by Ofte (2002) reported that males performed significantly better than females in overall correct scores. In contrast, our sample did not show a significant main effect of sex on BLRDT performance metrics. The absence of this sex difference in our objective measure, while contrasting with the original results, is not unique in the literature, which has shown divergent findings regarding sex differences in objective LRC performance (Hanna, 1990; Snyder, 1991). The successful replication of the Arm Position effect, however, validates that the underlying mechanism of spatial perspective-taking was reliably captured in our sample, providing a robust basis for the subsequent analysis of the NEST paradigm.

Similarly, the LRCT showed a significant increase in RTs corresponding to the increasing complexity of the verbal command. This reflects the known strain on verbal-semantic memory and language processing required for executing more complex LRC instructions. The successful replication of these core effects (consistent with Ocklenburg et al., 2011; Hirnstein et al., 2009) validates that our sample exhibits typical cognitive processing mechanisms and provides a robust benchmark for evaluating the novel NEST paradigm. However, this replication primarily establishes the cognitive competence of our general sample; it does not eliminate the need for future validation with the target population, who may process spatial information differently due to expertise and occupational stress.

The analysis of the subjective LRQ provided further evidence of replication, particularly concerning the established sex difference in self-reported LRC. Historically, self-reported studies have consistently indicated that females find themselves having more trouble distinguishing left and right than males (Hannay et al., 1990; Manga and Ballesteros, 1987; Snyder, 1991; Williams et al., 1993; Wolf, 1973). Consistent with previous literature, women reported significantly greater overall scores than men. This effect was primarily driven by the LRC dimension, while the DIR dimension showed only a marginal difference. Moreover, the expected hierarchical effect within the LRQ was replicated: participants reported significantly higher scores for the DIR items than for the LRC items (Jordan et al., 2006; Ocklenburg et al., 2011).

### 4.3 Validation and ecological complexity of the NEST (H2)

The findings from the Never Events in Surgery Test (NEST) demonstrate its ecological complexity and context-dependence, confirming our H2. Performance (both error rates and RTs) varied significantly depending on the posture of the training doll. Notably, error rates were significantly lower in the prone condition compared to the left and right lateral conditions (*p* < 0.05). This finding aligns with studies demonstrating that spatial alignment is critical to LRC performance, specifically reporting that facing the stimuli's backside (a scenario analog to the prone position) increases accuracy by facilitating side congruency (van der Ham et al., 2021). This suggests that a body-proximal, easily visualizable orientation (like prone, which often permits simple side-assignment) reduces LRC, whereas lateral positions, requiring a more complex spatial shift and integration of the operator's own frame of reference, induce more errors (McKinley et al., 2015). Regarding the supine position, error rates were intermediate and did not differ significantly from the prone position, suggesting that the axial mirroring inherent in this posture does not necessarily translate into a higher error propensity compared to the geometrically simpler prone position.

In contrast, the analysis of RTs revealed that the supine position yielded the shortest processing times (M = 3.96 s, SD = 1.34 s), significantly outperforming all other orientations (all *p* < 0.001). This is a remarkable finding since the supine position is the clinical standard, routinely used for anesthesia induction and a wide range of surgical procedures (Phillips and Hornacky, 2020, S. 479-513). Also, one might expect the supine position to impose a high cognitive load due to the required 180° mental rotation and the inversion of the left-right axis (McKinley et al., 2015). However, our data suggest that the supine position provides the most efficient cognitive access. This advantage could stem from the high visibility of ventral anatomical landmarks (e.g., facial features, navel), which serve as salient spatial anchors for rapid orientation. In addition, from a methodological perspective, the supine setup in the NEST may afford comparatively shorter, more linear pointing trajectories along the patient's sagittal axis, whereas lateral postures impose more oblique movement paths and a stronger alignment between the participant's own body axis and the patient's orientation, thereby incurring additional temporal costs despite comparable error rates.

### 4.4 Relationship of sex and LRC performance in all examined tasks (H3)

Crucially, the anticipated sex differences (H3), frequently reported in spatial tasks like the BLRDT, were not observed in the NEST. There was no significant main effect of sex nor a sex × condition interaction for either error rates or RTs. This outcome is highly relevant, as it suggests that the added, applied processing requirements of the NEST (integrating auditory instruction, positional changes, and embodied action) may override the typical sex differences observed in purely mental rotation tasks. Only in the subjective self-assessment, the LRQ in the LRC category, did women rate themselves significantly lower than men. In the various objective measurements, however, there was no significant difference in this sample.

### 4.5 Cognitive dissociation: error rates vs. reaction Time (H4)

The central contribution of this study, and the confirmation of our Hypothesis H4, lies in the dissociation between error propensity and processing speed across the basal and applied tasks.

The multiple linear regression model predicting NEST error rates from the established tasks (BLRDT_errors_, LRCT_errors_, LRQ_scores_) was not significant (R^2^ adj. = −0.026, *p* = 0.746). This is a critical finding: It strongly suggests that LRC susceptibility in applied contexts, as measured by the NEST, constitutes a largely independent risk factor that is not fully captured by traditional psychometric assessments of spatial-cognitive or verbal-semantic LRC components. However, regarding our a priori expectations, it must be noted that this hypothesis was only partially supported. While the non-significant regression model confirms that baseline metrics do not majorly predict NEST variance, the significant bivariate error correlations between the NEST and the baseline tasks (LRCT and BLRDT) ran counter to our original hypothesis of non-significance. This indicates a small but statistically significant shared variance that prevents the NEST from being interpreted as a completely isolated measure. Errors in the surgical environment may not stem solely from a general weakness in mental rotation or verbal labeling, but rather from specific failures in perspective integration and action planning under ecological stress.

A further critical finding that underpins the necessity of context-specific measurements is the presence of extreme individual differences in LRC susceptibility. Independent of the failure of the basal tests to predict errors, several participants in the NEST exhibited conspicuously high error rates, aligning our sample with previous research findings. Yamashita (2022) demonstrated in a large sample that there are neurologically intact individuals who exhibit extreme LRC both subjectively and objectively. This observation is crucially important: it suggests that the high-error outliers observed in NEST are not merely statistical artifacts but rather reflect a highly susceptible subpopulation. The NEST thus proves to be a sensitive indicator that can reliably identify the presence of these at-risk individuals in a simulated clinical setting, even when the overall group error rate is low.

In sharp contrast, LRCT reaction time significantly and robustly predicted mean NEST reaction time (R^2^ adj. = 0.348, *p* < 0.001). This robust correlation indicates that the tasks share a common component of general cognitive processing speed (Jensen, 2006). While the speed of information processing remains consistent across contexts, it is uniquely optimized in the supine position (*M* = 3.96 *s*). Despite the theoretical requirement for a 180° axis-transformation, participants processed commands in this orientation most rapidly, suggesting that salient anatomical landmarks in the supine view facilitate a more efficient transition to an allocentric reference frame compared to the more ‘feature-poor' prone or lateral orientations. Besides that, error vulnerability is uniquely modulated by the applied demands of the ecologically complex NEST setting (auditory command, physical action, shifting perspectives). By analyzing only correct trials, we ensured that the observed correlation reflects actual cognitive processing speed rather than being confounded by trial-and-error behavior or measurement noise from incorrect responses.

### 4.6 Limitations

We utilized a fixed, non-randomized order of tasks (BLRDT, LRCT, NEST) to ensure that basal cognitive measures were assessed prior to the complex, applied task. While this decision was theoretically motivated to avoid carryover effects from the highly complex NEST back onto the basal tasks, it introduces a potential confounding factor: the impact of fatigue or practice effects on the subsequent tests. Future studies should focus on utilizing a fully counterbalanced design to mitigate the potential effects of the fixed task order.

The current sample was primarily composed of university-affiliated participants with a relatively young mean age (*M* = 27.7 years). These demographic limits the generalizability of the findings to the actual target population (health professionals) who operate under chronic stress and professional familiarity. Future research must validate the NEST paradigm with medical professionals and assess whether high professional expertise moderates the observed dissociation between basal and LRC performance in applied contexts.

As discussed in Section 4.4, the generalizability of our findings must be considered in light of certain sample characteristics. Notably, the objective LRC measures (BLRDT and LRCT) did not reveal the sex differences typically reported in the literature, where women tend to exhibit lower performance on objective left-right discrimination tasks (Hirnstein et al., 2009; Hjelmervik et al., 2015; Ocklenburg et al., 2011; Ofte and Hugdahl, 2002). In our sample, sex differences were only evident in the subjective self-assessment (LRQ), where women rated their own LRC ability as significantly lower. This unusual pattern across the objective measures suggests a potential issue regarding the representativeness of the sample concerning general LRC aptitude and warrants caution when interpreting the absolute performance levels.

The experimenter manually recorded error scoring into the software for the NEST, which, despite the use of video recording for verification, could introduce subtle observer bias or measurement latency compared to purely automated systems. Similarly, a methodological constraint regarding the operationalization of reaction times in the NEST must be acknowledged. Unlike fully computerized benchmark tasks where the user provides direct digital input, the physical nature of the NEST required manual trial termination. Although PsychoPy automatically logged and saved the reaction times into the data file at the end of each trial, the timer was stopped via a keyboard press by the experimenter the exact moment the participant executed their pointing gesture on the training doll. While this procedure successfully accommodated the tactile, embodied setup without necessitating complex motion-capture sensors, it implies that the recorded RTs include a minor component of experimenter latency. However, because the same registration procedure was applied consistently across all participants and trials, this source of measurement error is unlikely to have introduced systematic bias in individual differences.

A methodological limitation concerns the utterance speed of the verbal commands in both the LRCT and the NEST. The commands, generated using the text-to-speech program *Balabolka*, were perceived by participants as being delivered too slowly. Some participants explicitly reported that the prolonged duration of the spoken command itself provided them with ample time for extensive deliberation before the instruction was fully conveyed, allowing them to consciously avoid errors. We posit that this slower-than-optimal articulation rate likely contributed to the lower-than-expected error rates observed, particularly in the LRCT. For future research and further development of the NEST, increasing the speed of the articulation rate would be crucial. A faster, more time-pressured command delivery would heighten the cognitive load and increase the ecological validity of the tasks, better reflecting the rapid, high-stakes decision-making environment of the operating theater, and could potentially reveal a more pronounced error propensity in both the LRCT and the NEST.

Another methodological constraint of the present study is the fixed administrative order of the tasks (BLRDT, followed by LRCT, and finally NEST). This sequence was originally chosen to ensure that basal cognitive measures were captured prior to the complex simulated paradigm. However, this fixed order introduces potential confounding sequence effects. For instance, completing two consecutive left-right discrimination tasks could cause a priming or practice effect, potentially inflating performance on the subsequent NEST and attenuating error-rate correlations. Conversely, cognitive fatigue toward the end of the session might have introduced unsystematic noise into the NEST error rates, which could artificially weaken the observed associations with basal measures. Consequently, the lack of counterbalancing limits the robustness of our dissociation claim. Furthermore, the posture order of the simulated training doll within the NEST paradigm was fixed for all participants. This lack of counterbalancing implies that intra-task learning effects (e.g., progressive adaptation to spatial rotations) or cognitive fatigue could have influenced performance on later postures. To address this limitation, future validation studies should, and in our ongoing follow-up research already do, utilize fully randomized task sequences to systematically rule out order-induced artifacts.

Finally, a notable limitation of the present study is that the NEST paradigm was evaluated strictly at the individual level. In clinical practice, surgical time-outs and procedures are performed within a multidisciplinary team environment, where group dynamics, roles, and communication styles heavily influence error-trapping. Future research should investigate how individual LRC and NEST scores translate into group performance. Specifically, it remains to be determined whether certain individuals with high LRC have a disproportionate impact on a team's collective decision-making, and how factors such as team roles or sex composition modulate these effects in simulated group environments.

### 4.7 Implications for practice and patient safety

The results have direct and critical implications for patient safety and the prevention of wrong-site surgery. Since NEST error rates are not predicted by established LRC measures, screening surgical staff for general LRC ability is insufficient, due to traditional basal tests failing to capture the context-specific, applied cognitive load that leads to operational errors in the surgical environment. Prevention strategies must instead focus on the context-dependent and ecologically specific error sources modeled by paradigms like NEST. This demand for context-specific solutions and rigorous quality management aligns with the institutional requirements for all high reliability organizations in the healthcare sector, where preventable failures in complex environments must be addressed through targeted, systemic measures (Maurer et al., 2025).

Consequently, given that the ability to distinguish between left and right is known to vary significantly among medical students (Gormley et al., 2008), the NEST or similar paradigms should be integrated into surgical simulation training to specifically cultivate the ability to perform accurate perspective integration in the operational context.

The finding that NEST performance significantly depends on patient posture and operator perspective underscores the need for surgical site marking, Team-Time-Outs and checklists (Fudickar et al., 2012) to explicitly incorporate the patient's physical position into the side verification process (Panesar et al., 2011). In situations where anatomical landmarks are less visible or axes are congruent but unfamiliar (e.g., prone or lateral positions), reaction times increase, indicating higher cognitive mapping effort. However, the supine position poses a different risk: the high processing speed (3.96 *s*) in this most common surgical orientation might mask the underlying axial incongruency. This could lead to ‘fast but wrong' decisions if the 180° transformation is not consciously verified. Therefore, verbalizing the perspective (e.g., ‘Patient is supine, viewed from the foot') could be essential to align the team's mental reference frame with the rapid but potentially error-prone automated response. This paradigm shift moves verification from a simple check of the limb to an active consideration of the mental transformation required by the surgical team.

Our results position LRC susceptibility in an applied context as a largely independent risk factor for laterality errors. Developing screening tools based on NEST principles could help identify individuals with a high propensity for context-dependent errors and support them with targeted situation-specific training.

### 4.8 Conclusion

The present study contributes to patient safety by introducing and validating the NEST, thereby closing a critical gap in the assessment of LRC for applied contexts. Our central finding establishes a profound cognitive dissociation between processing speed and error propensity in basal vs. applied LRC tasks. On the one hand, NEST RTs significantly correlated with those of the LRCT, reflecting a robust, context-spanning component of general cognitive processing speed. On the other hand, the regression model utilizing the error rates of the established psychometric measures (BLRDT, LRCT, and LRQ) failed to demonstrate any significant predictive power for the error rate observed in the NEST.

This crucial error dissociation suggests that vulnerability to LRC in an applied context does not stem from a general weakness in mental rotation or verbal labeling but constitutes a largely independent, context-specific risk factor. Error susceptibility is thus uniquely modulated by the complex, applied requirements of perspective integration and action planning under ecological stress. This was further substantiated by the NEST's validation, which showed high ecological sensitivity: performance significantly varied depending on the posture of the training doll. Furthermore, we found no significant sex differences in either error or RT data in this applied task, indicating that the additional procedural demands of the NEST may override the typical sex differences found in purely mental spatial tasks.

In summary, these results demonstrate that the LRC susceptibility in an applied context measured by the NEST is a largely independent, context-specific cognitive risk factor for laterality errors that is not fully predicted by standard measures. They demonstrate that the prevention of wrong-site surgery requires a paradigm shift in research and practice, moving away from general LRC screening toward tailored, context-specific approaches. The NEST paradigm, due to its high ecological validity and capacity to measure errors under time-critical, multi-perspective load, provides an essential foundational behavioral instrument that offers the potential to assess these operational risks.

## Data Availability

The datasets presented in this study can be found in online repositories. The names of the repository/repositories and accession number(s) can be found below: https://osf.io/3a6yz?view_only=36c971a0c6974c799652222783083d03.
